# Self-Generated Hypoxia Leads to Oxidative Stress and Massive Death in *Ustilago maydis* Populations under Extreme Starvation and Oxygen-Limited Conditions

**DOI:** 10.3390/jof7020092

**Published:** 2021-01-28

**Authors:** Jelena Petkovic, Milorad Kojic, Mira Milisavljevic

**Affiliations:** Institute of Molecular Genetics and Genetic Engineering, University of Belgrade, Vojvode Stepe 444a, 11042 Belgrade, Serbia; jelena.petkovic@imgge.bg.ac.rs (J.P.); mdkojic@imgge.bg.ac.rs (M.K.)

**Keywords:** water-transfer, hypoxia, oxidative stress, starvation, persistent cells

## Abstract

*Ustilago maydis* and *Saccharomyces cerevisiae* differ considerably in their response to water-transfer treatments. When stationary phase cells were transferred to pure water and incubated under limited supply of oxygen, the *U. maydis* cells suffered a catastrophic loss of viability while the *S. cerevisiae* population was virtually unaffected by the treatment. The major factor underlying the death of the *U. maydis* cells under those conditions was an oxygen-consuming cellular activity that generated a hypoxic environment, thereby inducing oxidative stress and accumulation of reactive oxygen species, which resulted in lethality. Importantly, a small residue of *U. maydis* cells that did survive was able to resume growth and repopulate up to the initial culture density when sufficient aeration was restored. The regrowth was dependent on the cellular factors (Adr1, Did4, Kel1, and Tbp1), previously identified as required for repopulation, after killing with hydrogen peroxide. Surprisingly, the survivors were also able to resume growth under apparently hypoxic conditions, indicating that these remnant cells likely switched to a fermentative mode of growth. We discuss the findings in terms of their possible relevance to the eco-evolutionary adaptation of *U. maydis* to risky environments.

## 1. Introduction

To survive, remain active, and reproduce in natural environments, microorganisms must be able to detect and respond fittingly to a myriad of external variables that might challenge their intracellular homeostasis, physiology, and genome integrity. These environmental variables include a wide spectrum of factors, ranging from the fluctuations in the types and quantities of available nutrients through the occurrence of adverse conditions such as elevated temperatures, desiccation, flooding, high-osmolarity, acidity, etc., to the presence of very pernicious agents that are different forms of irradiation and toxic compounds. Within this remarkable range of factors, nutrient starvation might well be the most common stress experienced by microorganisms in their natural habitats, and many excellent lab-studies were done exploring this problem within different conceptual and experimental frameworks, including the most extreme form of starvation, namely “water-transfer” [1]—a term used to refer to the transfer of cells from a growth medium to pure water.

When stationary-phase *Saccharomyces cerevisiae* cells are transferred and incubated in water they remain viable for weeks [2,3]. Actually, it was shown that this transfer extends the yeast chronological lifespan [4,5] and that the extension is dependent on the serine/threonine kinase Rim15 and other components of the RAS and TOR pathways [6]. It was also demonstrated that autophagy is upregulated in water-transferred yeast, and that it is required for chronological lifespan extension, upon transfer [7]. Furthermore, the maintenance of cell viability under this extreme starvation is also dependent on proteasomes and on the ongoing protein synthesis of a limited set of, presumably, the most essential proteins [1]. Generally, this same study revealed that in response to water-transfer, yeast cells mount a complex response that, through certain reorganizations of the intracellular milieu, results in a prolonged lifespan, by maintaining the characteristics of young yeast. In any case, yeast cells stop dividing immediately upon water-transfer [1]. However, it should be recalled that if the water-transferred yeast cells are incubated in the presence of either fermentable or non-fermentable sugars, morphological and physiological changes characteristic of mitotically growing cells are induced, causing a drastic loss of viability within a few hours [2,3]. Namely, under extreme starvation conditions, addition of sugars, signals the presence of sufficient nutrients for growth, yet in the absence of the complementing nutrients to support growth, the pre-budding growth events are followed by the production of reactive oxygen species (ROS), leading to apoptotic death [8].

Recently, we have become interested in investigating the ability of *Ustilago maydis*, a yeast-like basidiomycotan fungus for recovery from heavy, peroxide-induced oxidative stress, under non-nutrient (practically, under water-transfer) conditions. As reported in [9], the approach revealed that following massive oxidative damage, the fungus can, even in water, reconstitute its decimated populations by reproduction of the survivors, at the expense of the dead or dying cells. The phenomenon was termed “regrowth (or repopulation) under starvation (RUS)”, and was studied further by involving *S. cerevisiae* and using a naturally occurring stressor, desiccation [10]. Therefore, the comparative study we conducted found that although both organisms reconstitute devastated populations through the same process, there are significant differences between them that result in the relative superiority of *U. maydis* cells to perform RUS. The main reasons underpinning this superiority are—(1) evidently faster decomposition and leakage of the intracellular biomolecules and (2) a greater ability of *U. maydis* cells to recycle damaged and released organic materials. Interestingly, the most prominent difference in the post-stress degradation of intracellular macromolecules was observed in the case of genomic DNA. Following peroxide treatment or desiccation, the chromosomal DNA is abruptly and rapidly degraded in *U. maydis*, but is scarcely affected in the dying *S. cerevisiae* cells [10].

However, in the course of these comparative studies, while considering the effects of cell density, buffers, or inorganic salts, on the regrowth rate and final yield in the treated cell populations, we noted another distinctive difference between *U. maydis* and *S. cerevisiae* that was manifested only in high cell density controls. Namely, when *U. maydis* and *S. cerevisiae* cells were incubated in water at high densities (10^8^ cells/mL), the cultures of *U. maydis* underwent catastrophic cell death within just 1 day of treatment, while the viability of *S. cerevisiae* remained virtually unaffected during at least 4 days of incubation. This unsuspected observation provoked a number of thoughts for us to consider. First, this simple result suggested that there must be some (unknown) fundamental difference in the starvation-biology between these two evolutionarily diverged fungal species. Second, this intriguing difference might reflect the differences in the environmental conditions that shaped the life of these fungi over evolutionary time. Therefore, understanding why *U. maydis* and *S. cerevisiae* behave differently under these conditions seemed to be an important problem worth investigating. Thus, driven by these considerations, we decided to further investigate the reasons underpinning the massive death observed in starving/water-transferred *U. maydis* cell populations. Described below are experiments aimed at that direction.

## 2. Materials and Methods

### 2.1. Organisms and Culture Media

Water-transfer experiments were conducted using wild-type haploid prototroph *U. maydis* 521 strain (originally obtained from Professor Robin Holliday) and wild-type haploid prototroph *S. cerevisiae* ML1131 (kindly provided by Professor Michael Lisby, University of Copenhagen, Denmark). *U. maydis* was grown in YEPS (1% yeast extract, 2% peptone, and 2% sucrose) and *S. cerevisiae* was propagated in YPD (1% yeast extract, 2% peptone, and 2% glucose). Since the RUS mutants used in this study were derivatives of the *U. maydis* UCM520 (*nar1-1 met1-2 a2 b2*) [11], this strain was used as the nominal wild-type control for the analyses of the usability of the substrates derived from dead or dying cells. *met, nar, a,* and *b* indicate auxotrophic requirement for methionine, inability to utilize nitrate, and mating type loci, respectively.

### 2.2. Analysis of Water-Transfer Survival

Cells were grown at 30 °C in loosely-capped bottles, with constant shaking (200 rpm) to an early-stationary phase, generally for 16–20 h of incubation, when the cultures would reach approximately 2–4 × 10^8^ cells/mL. The cells were pelleted at 4000× *g*, washed twice in an equal volume of sterile, double-distilled water, and resuspended in water at the indicated cell densities. If not specified otherwise, the washed cells were incubated as 1 mL suspensions in 1.5 mL tubes at 30 °C, with shaking. The tubes were sealed by parafilm. To determine cell viability, aliquots were taken at the times indicated, diluted appropriately, and plated on the YEPS plates. The YEPS plates were incubated for 2 days at 30 °C for colony formation. The water used in these experiments was purified by a Reverse Osmosis Water System (C.C.K. Taiwan). Atmospheric oxygen was exchanged with ultra-pure N_2_ gas (Messer Tehnogas, Serbia).

### 2.3. Analysis of ROS in Water-Transferred U. maydis Cells

Determination of ROS level was performed according to [12]. After incubation of cells in water for the indicated times (12 h, 16 h, 18 h, and 24 h), cell density was adjusted to 4 × 10^6^ cells per ml and 100 µL was mixed with 2,7-dichlorofluorescein diacetate (DCF-DA) (Life Technologies, Oregon, USA), to a final concentration of 100 µM. Cell suspensions were incubated for 30 min at 37 °C in the dark, and fluorescence was measured at 480 nm excitation and 530 nm emission in 96-well microtiter plates (BioLite 96 Well Multidish, ThermoFisher Scientific, NY, USA) in a Plate Reader Infinite M200 PRO, Tecan, Austria GmbH. All experiments were repeated at least three times. Statistical analysis of ROS production in hypoxia over time was tested by the analysis of variance (ANOVA) model. The significance of differences among sample means was analyzed by the post-hoc Tukey test at a significance level of 0.05, using the statistical software SPSS 21.0 (IBM, Armonk, NY, USA).

### 2.4. DNA Extraction and Gel Electrophoresis

Chromosomal DNA was extracted from cells immediately upon transfer into water and at various time-points, after 24 h of incubation at 30 °C, according to the method described by Doyle and Doyle (1987) [13], with some modifications. In brief, 4 × 10^7^ cells were suspended in 800 µL buffer containing 2% CTAB, 100 mM Tris-HCl, pH 8.0, 20 mM EDTA, 1.4 M NaCl, 2% polyvinylpyrrolidone, and mixed with ~100 µL glass beads (~0.5 mm). After vigorous agitation on a vortex mixer samples, DNA was extracted with 800 µL chloroform:isoamyl alcohol (24:1). Phases were separated by centrifugation and the aqueous phase was extracted again. Nucleic acids were precipitated with ethanol and the precipitates were washed with 70% ethanol. After removal of ethanol, DNA was redissolved in 30 µL RNaseA solution (20 µg per mL in water) and incubated for 10 min at 37 °C. The DNA was resolved in 1% agarose gels containing 0.5 µg/mL ethidium bromide.

### 2.5. Propidium Iodide (PI) Staining of U. maydis Cells

A total of 4 × 10^6^ cells per mL were resuspended in 200 µL of PBS buffer (1.37 M NaCl, 27 mM KCl, 100 mM Na_2_HPO_4_, 18 mM KH_2_PO_4_) and PI was added to the final concentration of 100 ng/µL, and incubated for 30 min, at 30 °C in the dark. Cells were placed on a glass slide with a glass coverslip and observed under a fluorescent microscope BX51 (Olympus, Tokyo, Japan), with a ×10 objective, using a filter block with excitation 530–570 nm.

## 3. Results

### 3.1. Cell Density-Dependent Loss of Viability in U. maydis Populations

**Figure 1:**
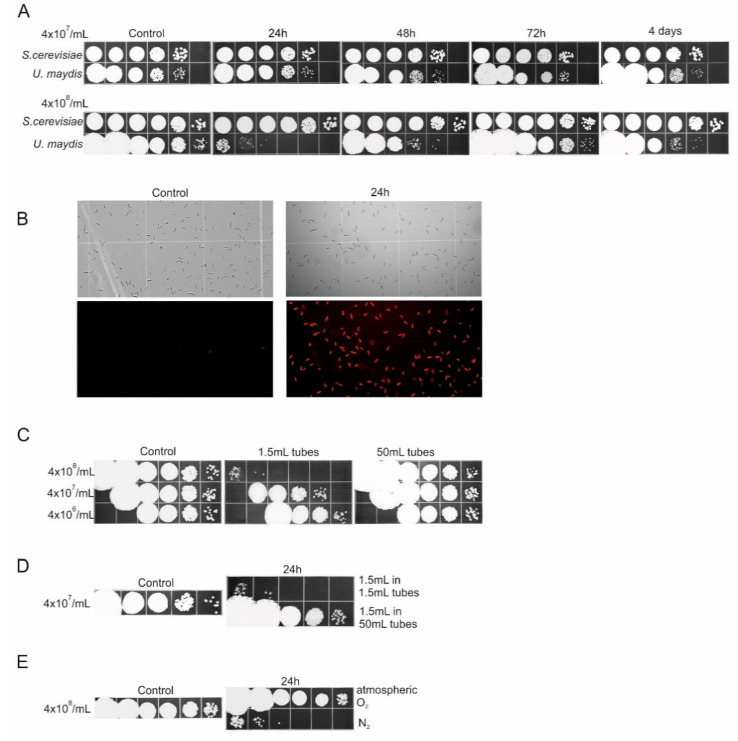
Viability of *S. cerevisiae* and *U. maydis* cells upon water transfer. (**A**) One milliliter of cell suspensions of 4 × 10^7^ cells/mL (upper panels) and 4 × 10^8^ cells/mL (lower panels) were incubated in water in 1.5 mL tubes for 4 days. Aliquots were withdrawn at one day intervals, used for preparation of 10-fold serial dilutions, and spotted on the solid rich medium. (**B**) Brightfield and fluorescent imaging of cells stained with propidium iodide (PI). Water-transferred early-stationary phase cells (4 × 10^8^ cells/mL) were stained with PI before (Control, left micrographs) and after 24 h of incubation (right micrographs) for detection of damaged/dead cells. Fluorescence micrograph of 24-h-incubated cells clearly showed that nearly all cells were fluorescing red, indicating membrane damage. (**C**) One milliliter of *U. maydis* cell suspensions (4 × 10^8^/mL, 4 × 10^7^/mL, and 4 × 10^6^/mL) were incubated in water in 1.5 mL tubes and 50 mL tubes for 24 h. Ten-fold serial dilutions were spotted on the solid growth medium. (**D**) One-and-a-half milliliter of *U. maydis* cell suspensions (4 × 10^7^/mL) were incubated in water for 24 h in 1.5 mL tubes and 50 mL tubes; 10-fold serial dilutions were spotted on the solid growth medium. (**E**) One milliliter of 4 × 10^8^ *U. maydis* cell suspensions were incubated in water in 50 mL tubes for 24 h, under atmospheric air (O_2_) or under N_2_ atmosphere. Ten-fold serial dilutions were spotted on the solid growth medium. Left panels represent the starting number of cells in suspensions (Control). All experiments were performed at least three times and the representative results are shown.

In a rich medium, cultures of *U. maydis* cells reach a density of about ~4 × 10^8^ per mL, after overnight growth, and exhibit little loss in viability over the course of several days, when stored at 4 °C. In preparation of cells for determining cell survival during RUS or treatment with DNA damaging agents, we routinely washed cells from fresh overnight cultures in water and resuspended them in water at a density of about 2–4 × 10^7^ per mL. We noted little, if any, decline in cell viability if 1 mL of these suspensions was incubated in 1.5 mL tubes at 30 °C, over the course of 4 days. It was, therefore, surprising to find that when cells were washed, resuspended in water at the original culture density of ~4 × 10^8^/mL and incubated under identical conditions, they underwent a massive death within 1 day of incubation (Figure 1A). In sharp contrast, *S. cerevisiae* did not show, under an equivalent setup, any appreciable loss of viability for up to 4 days of treatment (Figure 1A). In fact, no indication of lethality was observed in *S. cerevisiae*, even when the cells were incubated at a density of about 4 × 10^9^ per mL.

As the preceding results are based on determination of cell viability/survival by colony forming units, i.e., by their ability to reproduce, it is possible that the cells in the highly dense 24-h-incubated suspensions were viable, but their ability to form colonies was inhibited by rapid entry into some kind of dormant, non-growing state. Therefore, to test this somewhat remote possibility, we determined cell viability in the water-transferred suspensions, before (Figure 1B Control) and after 24 h-incubation, using propidium iodide staining, a well-established technique for distinguishing viable from nonviable cells. In sharp contrast to what was found for the control samples, nearly all of the 24-h-incubated cells showed a positive PI staining, confirming that the cells did, indeed, undergo cell death upon treatment. Thus, we could conclude that unlike *S. cerevisiae*, *U. maydis* exhibits some sort of “crowding effect”, resulting in a staggering 5-log drop in viability. Could it actually be that at high cell densities, *U. maydis* produces and emits some suicidal signal that mediates a catastrophic demise of the population?

Consequently, we intended to probe this interesting possibility but before proceeding in that direction we realized that for devising an appropriate methodological strategy, a higher resolution assessment of the kinetics of the survival of water-transferred *U. maydis* cells should first be obtained. Thus, we incubated a suspension of *U. maydis* cells (4 × 10^8^ per mL) under the same conditions as above and assayed it for viability, by withdrawing aliquots at every 4 h for 24 h. Surprisingly, no loss of viability was seen at any time-point over the entire period of the experiment. Clearly, the short periodic exposures to atmospheric air—at the points of taking the aliquots—were sufficient to completely inhibit the induction of mortality, ruling out the idea that the drastic loss of viability observed upon continuous 24-h-incubation was somehow induced by high cell density *per se*. In other words, reaeration at 4 h intervals was enough to sustain cell viability in high-density suspensions. Therefore, we inferred that the critical factor for the maintenance of viability was the availability of sufficient oxygen, i.e., an adequate ratio between the number of cells in the suspension and the quantity of available oxygen. Indeed, several lines of experimental evidence reinforced this argument. First, cell viability was virtually unaffected when 1 mL of a highly dense (4 × 10^8^ cells/mL) suspension was incubated in a 50 mL tube (Figure 1C). In a reciprocal experiment, incubation of 1.5 mL of a less dense (4 × 10^7^ cells/mL) suspension in 1.5 mL tubes, resulted in a precipitous loss of viability (Figure 1D). Finally, when the atmospheric oxygen in a 50 mL tube carrying 1 mL of a highly dense suspension was exchanged with N_2_, the culture underwent a catastrophic decline in viability after 1 day of incubation (Figure 1E), confirming that deprivation of oxygen was the causal factor that in water-transferred *U. maydis* populations led to cellular demise. To recap, high cell density *per se* could not be directly linked to the induction of mortality.

### 3.2. Water-Transferred U. maydis Cells Carry Out an Oxygen-Consuming Metabolism

**Figure 2:**
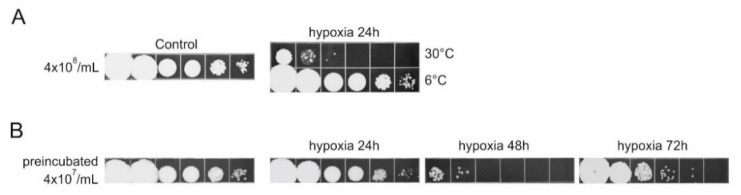
Oxygen-consuming metabolism affects cell survival under limited oxygen. (**A**) One milliliter of 4 × 10^8^ dense cell suspensions were incubated in water in 1.5 mL tubes at 6 °C or 30 °C, for 24 h; 10-fold serial dilutions were spotted on the solid growth medium. (**B**) Stationary cell culture, washed and brought to a density of 4 × 10^7^/mL was incubated in water for 24 h, prior to concentrating to a density of 4 × 10^8^/mL. One milliliter aliquots of high-density suspension were distributed in three 1.5 mL tubes and further incubated under limited aeration for 24 h, 48 h, or 72 h. At each time-point, suspension from a single tube was used for preparation of dilutions and were spotted on the solid medium. All experiments were performed at least three times and the representative results are shown.

All experiments described above support the view that the key condition for effective survival of water-transferred *U. maydis* cells is the availability of sufficient oxygen. Certainly, the observation that periodic reaeration of highly dense cell suspensions was sufficient to abrogate lethality could be taken as a strong indication that the water-transferred cells were metabolically active, that they respire, and consequently deplete the initial stock of oxygen, thereby, inducing cellular death. To strengthen this proposition further, we tested whether reduction of cell metabolism by two different conditions would postpone the decline of viability. First, we lowered metabolic activity by incubating the cells at a low temperature (Figure 2A). Indeed, slowing down cell metabolism by incubation of high cell density suspensions at 6 °C resulted in no decline of cell viability, in spite of the limited supply of oxygen. Second, to be metabolically active, besides oxygen cells need energy-rich nutrients and the only remaining source of energy-rich nutrients for the water-transferred cells is their own stock of organic reserves. Therefore, if water-transferred cells carry out an oxygen-consuming activity, prolonged incubation under sufficient aeration would lead to depletion of their reserves. If these preincubated cells are subsequently subjected to oxygen limited conditions, their survival would be expected to be more, extended relative to that observed when stationary phase cells are directly transferred to water. The experiment illustrated by Figure 2B was carried out precisely according to the logic just described. As expected, cells washed free of nutrients and preincubated in water at 2 × 10^7^ per ml for 24 h, with vigorous aeration, exhibited extended survival when concentrated and incubated under limited supply of oxygen. However, cell viability still declined within 48 h of incubation, indicating that the depletion of nutrients could only postpone but not completely abrogate the lethality. Taken together, our results indicate that in water-transferred *U. maydis* oxygen, presumably respiratory chain activity is required for the efficient maintenance of cellular viability. By implication, they also indicate that under conditions of limited stock of oxygen, the observed mortality was a consequence of self-generated hypoxia (SH), resulting from an oxygen-consuming cellular metabolism.

### 3.3. Temporal Survival Kinetics of Water-Transferred U. maydis Cells under Oxygen Limitation

In light of the observation that the decrease of viability in water-transferred *U. maydis* cell populations is caused by the self-generated hypoxia, a closer examination of the survival kinetics seemed even more relevant. However, given that the self-generated hypoxic conditions are gradually developed with the ongoing incubation and formed only if the starting supply of oxygen is limited, an obvious experimental difficulty was how to successively measure survival, without reaerating the samples. Thus, an adequate experiment required that for each time-point individual (single-use) aliquots were sampled and plated to measure viability.

**Figure 3:**
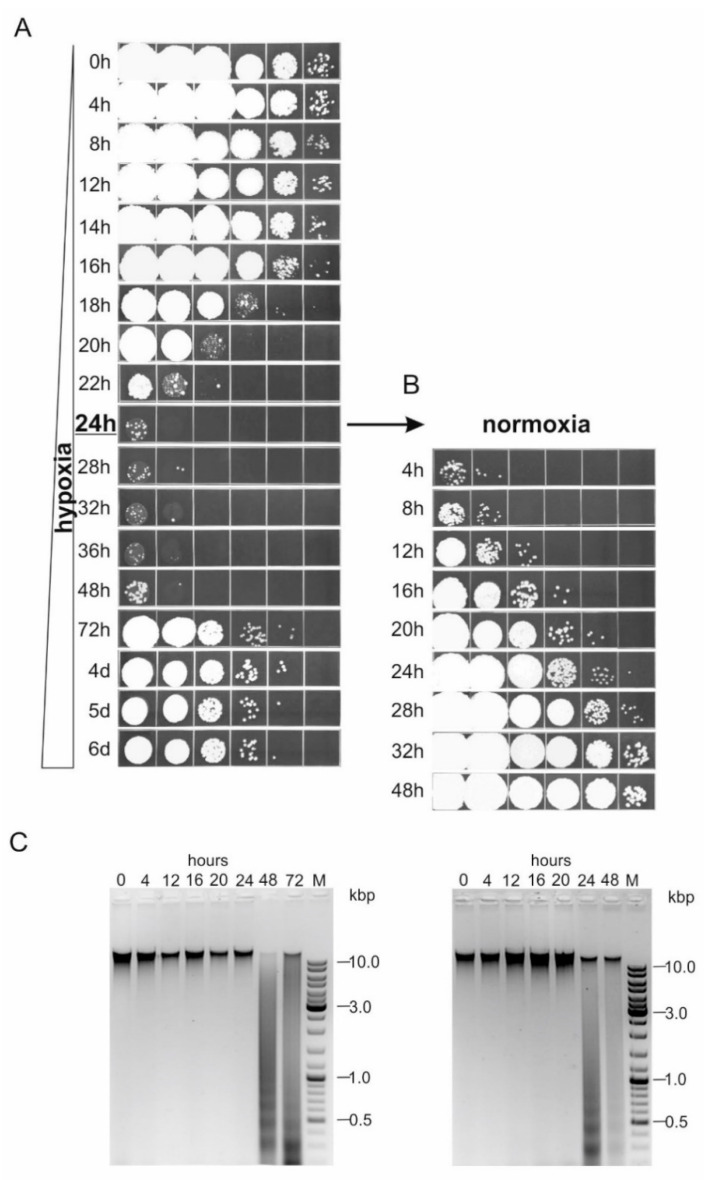
Survival kinetic of *U. maydis* high-density cell suspension during incubation in water under hypoxic and normoxic conditions. (**A**) 4 × 10^8^ cells/mL were incubated in water in 1.5 mL tightly sealed tubes, for induction of hypoxia. At the indicated time-points, a single tube was taken for preparation of 10-fold serial dilutions and spotted on the solid growth medium. (**B**) Cell suspension incubated in 1.5 mL tube for 24 h was further incubated in water in a 50-mL tube (normoxia) and dilutions were spotted at indicated time-points for monitoring of the recovery. (**C**) Agarose gel electrophoresis of genomic DNA extracted from cell suspensions incubated in water for the indicated time intervals under hypoxic (**left**) and normoxic conditions (**right**). M- GeneRuler DNA Ladder Mix, Thermo Scientific.

Therefore, the experiment was done in the following way. The cells harvested were washed twice with water and brought to a cell density of 4 × 10^8^ per mL. This suspension was carefully distributed in 1 mL aliquots into 1.5 mL tubes. Care was also taken to ensure that each of these tubes was tightly sealed by parafilm. The tubes were placed into a 96-well 1.5 mL microtube rack with lid, which was then mounted vertically on an oscillating shaker in a 30 °C room. At various time-points, these tubes were successively sampled to determine cell viability over the course of 6 days. Importantly, the survival kinetics obtained following this procedure were highly reproducible across replicate cultures, both in terms of timing and in terms of magnitude of change. Results shown in Figure 3A are representative of these determinations.

As can be seen, the number of viable cells remained fairly stable in the suspensions over the first 14 h of incubation. Thereafter, the cells rapidly began to lose their viability, dropping by more than four orders of magnitude within the next 10 h. This dramatic loss of viability was pretty remarkable but it is noteworthy that the population was not completely wiped out. A small residue of cells remained viable even after 48 h of incubation.

After 24 h of incubation, an aliquot was transferred in a 50 mL tube, so that the subsequent fate of the demised *U. maydis* cell populations was examined under both hypoxic (Figure 3A, left lower panels) and normoxic conditions (Figure 3B). This parallel monitoring of the devastated *U. maydis* cell populations revealed several intriguing observations. First, upon re-exposure to atmospheric oxygen, the number of viable cells increased exponentially, without any pronounced lag phase, reaching up to the level of the original suspension over the course of 32 h. On the other hand, and this was the most curious finding of this analysis, the small residue of viable cells could, after a 24-h period of adaptation, regrow under the very same environment that caused massive death, although yielding lower viable cell densities than those obtained in the aerated suspension. A likely possibility, thus, is that this tiny subpopulation of the remnant cells actually represents a ‘rump’ of the population that was capable of switching to a fermentative mode of growth. Why a significantly more robust repopulation was achieved upon re-exposure to atmospheric oxygen is a problem that cannot be resolved at the moment.

Another intriguing aspect of this analysis was the finding that the DNA exhibited quite different kinetics and pattern of degradation as compared to those observed upon damage imposed by peroxide or desiccation [10]. As can be seen in Figure 3C, chromosomal DNA showed little, if any degree of degradation over the first 24 h, and then was fragmented within the next 24 h, whereas in the peroxide-treated or desiccated *U. maydis* suspensions, DNA was abruptly and rapidly shattered completely into small fragments, within the first 12 h of incubation [10]. It is also very interesting to note that in contrast to what we observed in peroxide and desiccation experiments, we found a uniform and strong appearance of the fragmented DNA products. This DNA fragmentation pattern was reminiscent of the ordered fragmentation of genomic DNA (DNA laddering) that was found in many programed cell death (PCD) systems, as the result of DNA cleavage between nucleosomes [14,15,16]. Hence, these results might be taken as an indication that affected *U. maydis* populations might undergo different forms of death in response to different types and intensity of the stressogenic factors. Therefore, a detailed study of the mode of death induced by the self-imposed hypoxia would be of considerable interest.

### 3.4. The Increase in Viable Cell Densities Is Due to Multiplication of Surviving Cells Based on RUS-Dependent Recycling of Substrates Released from Dead Cells

The experiments described above all point fairly clearly toward the conclusion that water-transferred *U. maydis* is incapable of maintaining its viability if the initial stock of oxygen is limited. In particular, the vast majority of 24 h-incubated cells appear to be propidium iodide (PI)-positive, indicating cellular damage/death. A similar conclusion could be drawn from the time course analysis of the chromosomal DNA degradation. Therefore, it was logical to assume that the repopulation seen after the death-phase was promoted by multiplication of the survivors. Yet, strictly speaking, the results only support but do not prove the hypothesis. Namely, although remote, there is a formal possibility that PI-staining of the 24 h-incubated cells revealed only membrane damage but not cellular death. It was therefore necessary to ascertain whether the reconstitution of the devastated population was mediated by multiplication of the survivors or through intracellular repair (real recovery). This was done in an experiment using the experimental strategy we previously developed, to test the mode of recovery after killing with hydrogen-peroxide and desiccation [9,10].

**Figure 4:**
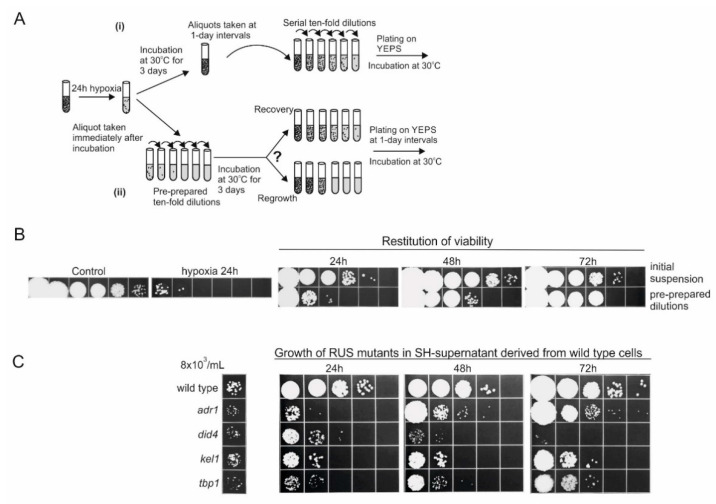
Restitution of viability under aeration of cells that died during incubation in water, in 1.5 mL tubes (24 h hypoxia). (**A**) Schematic diagram of the experimental design. The increase of cell viability was monitored at 1-day intervals in (i) the original suspensions of the cells incubated in water at 30 °C, under continuous agitation, before preparation of serial dilution and plating, or in (ii) pre-prepared 10-fold serial dilutions, following incubation under the same conditions. (**B**) Initial wild-type cell suspension (died upon 24 h-incubation under limited aeration) and its serial dilutions (pre-prepared dilutions) were incubated in water in 50-mL tubes under aeration, at 30 °C for 3 days, and spotted on the solid growth medium in one-day intervals. (**C**) Comparison of restitution of viability under aeration of wild-type and RUS mutants in supernatants derived from 24-h-incubated high cell density suspension of wt cells, under limited aeration (SH-supernatant). Wild-type cells (4 × 10^8^/mL) were incubated in water in 1.5 mL tubes (hypoxic conditions). After 24 h, cell suspensions were centrifuged and Millipore filtrated supernatants were inoculated with 8 × 10^3^ wild-type cells or RUS mutants, and incubated for the next 72 h under aeration (50 mL tubes). At one day intervals, aliquots were withdrawn, 10-fold serial dilutions were prepared and spotted on the solid medium. The panels are representatives of three independent experiments.

The experimental design is schematically presented in Figure 4A. Thus, in parallel to periodically taking aliquots from an undiluted suspension and subsequently making a serial of 10-fold dilutions (to be used for scoring the fraction of viable cells), we also monitored the viability in the pre-prepared 10-fold dilutions that were made immediately after the death-phase and incubated under the same conditions. Thus, if the restitution of viability is mediated through intracellular recovery, then the increase of the viable counts should in principle be uniformly seen in both series of dilutions. However, if the increase of viability is the result of cell multiplication, then in the series of pre-diluted suspensions, the growth that follows the incubation would strictly be evident only in the dilution tubes that contained viable cells at the moment of distribution. As shown in Figure 4B, the increase of colony forming ability was not equivalently present in both branches of the experimental setting, thus, one could conclude that no real recovery takes place and that the observed increase in viability was due to cell multiplication.

As ongoing multiplication of the survivors must rely on supply of energy-rich nutrients, which are clearly absent from water, it was logical to assume that the nutrients required for the re-growth must come from the dead or dying cells. To test this prediction, the 24-h cell suspensions were used to obtain cell-free supernatants, which were then inoculated with a fixed number (8 × 10^3^/mL) of fresh wild-type cells, and incubated at 30 °C with agitation. As shown in Figure 4C, the supernatant derived from 24-h suspensions, did contain nutrients in quantities sufficient to support numerous multiplications of the inocula (population size raises up to 1000 times), but not to the extent that was achieved in the re-aerated 24-h suspensions of cells (compare Figure 3B with Figure 4B). We assume that this difference simply reflects the fact that, as a function of time, the release of substrates from the dead or dying cells is not completed by the 24-h time-point. In other words, the ongoing reproduction of the survivors in the re-aerated 24-h suspensions of cells is most likely accompanied with further decomposition and continuing leakage of intracellular molecules from the dead cells, which in effect, can support a greater expansion of the growing subpopulation.

Since the genomic DNA fragmentation profiles suggested that the mode of death following peroxide-induced killing and SH-induced death might differ, and since cells dying by alternate forms of death release different (beneficial or harmful) products of cellular disintegration [17], into the suspending medium, it was of interest to determine whether the cellular factors involved in the regrowth after peroxide treatment were also required for reproduction on the substrates released by the SH-dying cells. Namely, in a previous report, we described the isolation of a number of RUS-deficient mutants by screening for the candidates that were unable to regrow in the suspension of peroxide-treated cells [9]. Four of them were characterized in considerable detail and found to be mutated in genes playing roles in growth regulation (adr1), protein turnover (did4), cytoskeleton structure (kel1), and transcription (tbp1). As after peroxide treatment, all four RUS mutants exhibited a dramatically reduced ability to reconstitute viability, following desiccation stress [10]. Thus, to test their ability to reproduce on the substrates released from cells dying from SH-induced death, we seeded 8 × 10^3^ cells/mL-inocula of each mutant, in the supernatant derived from 24 h-incubated high cell density suspensions, and the growth was examined at 1-day intervals. The results are given in Figure 4C. Clearly, in contrast to the wild-type, all RUS mutants exhibited a substantial reduction of growth in the medium removed from the 24 h-suspension, indicating that the common RUS factors were involved in the reconstitution of the devastated population, after killing by peroxide/desiccation and upon SH-induced death.

### 3.5. The Self-Generated Hypoxia Induces ROS-Production Which Is Causally Associated with Cell Death

**Figure 5:**
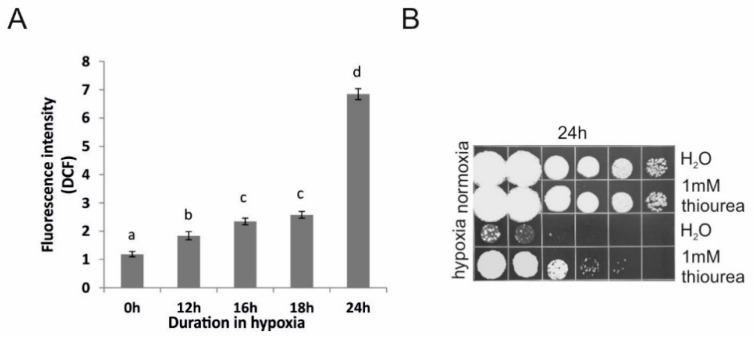
Production of ROS induce cell death under limited aeration. (**A**) ROS generation determined by measurement of DCF fluorescence. *U. maydis* cell suspensions (4 × 10^8^/mL) were incubated in water in 1.5 mL tightly sealed tubes for 12 h, 16 h, 18 h, and 24 h. For each time-point, a separate tube was designated. Error bars indicate standard deviations of four independent measurements. Significant differences (*p* < 0.05) between control cells and cells in hypoxia are depicted by different letters. (**B**) Survival of *U. maydis* high-dense cell suspension (4 × 10^8^/mL) incubated for 24 h in water and in the presence of 1 mM thiourea. Normoxia depicted incubation in loosely capped 50 mL tubes, and hypoxia in 1.5 mL tubes; 10-fold serial dilutions were spotted on the solid growth medium. Experiment was performed three times and the representative result is shown.

Lastly, we were interested in understanding how the self-driven hypoxia translated into cell killing. Given that hypoxia induced increased ROS-production in many experimental systems including fungi [18,19], and since the ROS accumulation is suggested to be the proximate cause affecting cellular death upon various types of stress [20,21,22,23], we examined whether the same was also true for the auto-produced hypoxia. Therefore, samples of cells kept at high cell density were taken at various time-points during incubation, so that the measurement of ROS production and intracellular accumulation was done before and during the viability decline. We measured the production of ROS using a commonly used fluorescent reporter-2′,7′-dichlorofluorescin diacetate (DCF-DA), which is converted into a fluorescent form after oxidation, which could be mediated by hydrogen peroxide and hydroxyl radicals [24]. The measurement revealed that *U. maydis* cells do, indeed, undergo an oxidative stress under our assay conditions. Namely, we detected a significant increase in the ROS levels, prior to the onset and during the death phase (Figure 5A).

Although the cell death was preceded by an increase in ROS production, it could in principle be that the result demonstrated only a positive correlation, not causation. Therefore, to test whether the observed production of ROS was causally associated with SH-induced cellular death, we treated cells with thiourea, a potent scavenger of hydroxyl radicals [25,26]. Indeed, we found that exposing *U. maydis* cells to 1-mM thiourea greatly (by >100 fold) improved their survival under our experimental conditions (Figure 5B). As a control for the toxicity of thiourea, the parallel suspensions of cells-exposed to 1-mM thiourea but incubated aerobically during the entire experiment—did not show any loss in cell viability (Figure 5B, upper panels). Thus, the result of this analysis strongly argues that ROS production plays a critical role in cell death induction. Therefore, we propose that SH results in oxidative stress and accumulation of toxic ROS to a level that triggers cell death processes, leading to a massive loss of viability in the *U. maydis* cells populations.

## 4. Discussion

The unexpected finding that initially prompted this work and around which the key conceptual and methodological facets of this study are organized, came from a rather simple experiment. When water-transferred *U. maydis* and *S. cerevisiae* cells were incubated at a high cell density and under a limited stock of oxygen, the population of *U. maydis* cells underwent a catastrophic loss of viability, whereas that of *S. cerevisiae* was virtually unaffected by the treatment. Importantly, after the crush, the devastated *U. maydis* population was able to reconstitute the initial level of viability (Figure 1A). The finding, thus, immediately indicated that there must be some fundamental physiological/biochemical differences that are associated with the cellular mechanisms employed by *U. maydis* and *S. cerevisiae*, upon water-transfer. Putting this into the eco-evolutionary perspective, the difference we observed might actually reflect specific survival strategies integrated in the different lifestyles of these two evolutionarily diverged fungal species and whose development was dictated by the particular requirements of their ecological niches.

Thus, this simple result suggested that there might be some hidden aspects of *U. maydis* biology worth being uncovered and further studied. Hence, we decided to extend these initial studies by focusing our investigation primarily on the underlying factors that might promote or mediate the induction of the massive death in water-transferred *U. maydis* cells. As a result, we uncovered a set of very intriguing facts about *U. maydis* water-transfer biology, but before plunging into the specifics of particular findings, it is worth remarking that only by applying the aforementioned experimental constraints, *U. maydis* was forced to reveal some of its secrets. Indeed, as hinted above, even the observation that served as the original impetus for this study came to light under these specific conditions. In addition, although it was found that hypoxia was the form of stress that mediated the induction of death, the events leading to and some of the processes following the death phase would not be readily revealed if we used the conventional methodological framework for directly studying the effect of hypoxia on *U. maydis* cells. These remarks, we should perhaps add, are not just side notes but an edited reflection that touch on the fact that there could be some elements of fortuity in this endeavor. In any case, the enquiry turned out to be quite revealing, producing some puzzling insights into *U. maydis* starvation biology and indicating a range of possibilities for further studies.

Thus, as we began to analyze the phenomenon more systematically, it soon became clear that the central underpinning point of difference between the water-transferred stationary phase *U. maydis* and *S. cerevisiae* cells, simply lies in the fact that *U. maydis* cells are metabolically active in water and that the fungus respires, i.e., that it consumes oxygen. Compared to the behavior of *S. cerevisiae* whose response to water transfer is characterized by a highly reduced metabolic activity (an energy preservation mode) and by an extended lifespan [1,27], the observation that *U. maydis* is metabolically active even when incubated in pure water seems rather curious. However, we are not in a position to comment further upon this intriguing finding, except to point out that a more comprehensive understanding of the overall “terrain” of *U. maydis* water-transfer physiology is needed, before the biological meaning of the differences between these two fungi can be grasped better.

Nevertheless, folded into this account is another puzzling aspect of this finding; if the supply of oxygen is limited, the metabolic activity leads to hypoxia and oxidative stress that results in massive cell death. Since the hypoxic environment was not externally imposed but developed by the cells themselves, it would appear that *U. maydis* is, for some reason, unable to halt the oxygen-consuming activity and thus to avoid reaching the “point of no return”, beyond which almost the entire population gets rapidly destroyed. Therefore, one is tempted to conclude that the death observed under our experimental conditions is a form of suicide. Given that a central belief in (micro)biology is that (micro)organisms can rapidly detect and promptly regulate the adequate response to environmental changes, it is reasonable to ask why does *U. maydis* fail to stop this self-defeating process? Indeed, what are the reasons due to which *U. maydis* exhibits suicidal response under our experimental conditions? Is this paradoxical behavior just an anomaly manifested due to the artificial setting of the experiment or is it an adequate reflection of an evolutionary ingrained behavior? In essence, could this self-imposed death be an adaptation?

This is well worth pondering, particularly in the context of the consistent observation that upon devastation, a residual number of viable *U. maydis* cells always remain. Importantly, these cells were able to resume growth and repopulate the devastated population when sufficient aeration was restored. This behavior is reminiscent of the phenomenon of non-heritable antibiotic persistence or phenotypic tolerance in bacteria, where slowed or arrested growth is suggested as a major factor underlying drug persistence [28,29]. Thus, it is tempting to propose that the small residue of cells, surviving in our experimental condition, actually represent persistent cells. It could, then, well be that the survivors are, in fact, slow-growing cells that are unaffected by hypoxia. This suggestion assumes even more importance when viewed in the light of the unexpected finding that these remnant cells were also capable of resuming growth under apparently hypoxic conditions. Accordingly, it seems logical to suppose that *U. maydis* might possess the genetic endowment that allows the fungus to switch to a fermentative mode of growth. If true, this would open up the question of whether this mode of growth is important for corn tissue invasion, during the parasitic stage? Along this line, recent findings linked the capacity of fungal pathogens to infect and colonize their mammalian hosts, with the ability of these fungi to adapt to various levels of hypoxia [30]. Interestingly, the studies identified ethanol in the tissues infected by the obligate aerobes [30,31].

Finally, the question arises of what this means in an ecological context? In its saprophytic form, *U. maydis* occurs in soil where it could periodically experience starvation. Since the availability of sufficient oxygen is the key to effective survival of *U. maydis* cells facing starvation, the conditions might become especially serious during periods of flooding when the soil becomes depleted in available oxygen. As gases, particularly oxygen, diffuse 10^4^ times more slowly through water than in air [32], a major constraint resulting from excess water is an inadequate supply of oxygen to aerobic microbes. In this scenario, we can entertain the possibility that the vast majority of the affected *U. maydis* population might rapidly die but rare, slow-growing cells survive. This minimal fraction of cells can subsequently switch to the regular, fast-dividing growth, thereby reconstituting the population after restoration of the oxygen supply. Thus, the phenomenon might have evolved to promote adaptation to environments characterized by episodes of catastrophic (flood) events.

## 5. Conclusions

Overall, in this report, we described the discovery of a rather interesting phenomenon related to the dynamics of water-transferred *U. maydis* cells, under a limited stock of available oxygen. The phenomenon was characterized by two dominant features, the processes of self-imposed catastrophe and rescue. Both of these processes have their own puzzling aspects, each deserving a much more extensive experimentation. Perhaps the most remarkable finding in this study is that the residue of viable cells can repopulate under the very same conditions that caused massive death. This unsuspected observation clearly underscored the polyvalent capacity of *U. maydis* to reconstitute the population in the aftermath of catastrophic environmental events. Together, our results foster the idea that *U. maydis* could have evolved a strategy for maximizing its long-term fitness that involves production of persistent cells and by this tactic, divides the risk among physiologically heterogeneous subpopulations. Under hospitable environmental conditions, the majority of *U. maydis* cells implement the prudent strategy of rapid growth and reproduction, whereas a tiny subpopulation of cells adopt the slow-growing “persister” state, which would repopulate if the environment changed. The population fitness advantage conferred by this strategy would, thus, be based on the degradation and release (by massively dying cells) and re-usage (by persister/surviving cells) of the degraded biomolecules. Clearly, the survivors could derive large benefits, but that must be coupled with efficient exploitation of the nutrients to outcompete the competitors [33].
